# Supplementary material for: E-Cadherin Modulation and Inter-Cellular Trafficking in Tubular Gastric Adenocarcinoma: A High-Resolution Microscopy Pilot Study
Source: Biomedicines. 2022 Feb 1;10(2):349. doi: 10.3390/biomedicines10020349 (PMC8961786; doi:10.3390/biomedicines10020349)
Supplement: Supplementary file 1 [file biomedicines-10-00349-s001.zip › biomedicines-1576239-supplementary.pdf]

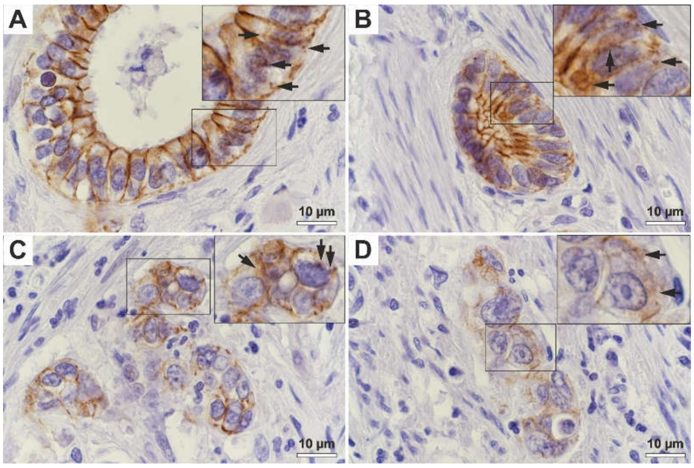

Supplementary Figure S1. Exemplary high magnification images of E-cadherin immunostaining in gastric tumors. (A,B) Well differentiated tubular adenocarcinoma presents with a preserved membrane expression of E-cadherin, and with intracytoplasmic dense-diffuse granule present (arrows). (C,D) Solid, poorly differentiated adenocarcinoma exhibit a reduced overall E-cadherin immunoexpression, however the dense-diffuse intracytoplasmic signal is still present in the cytoplasm and around the nucleus (arrows). The rectangles in each image are enlarged in the same frame; scale bars represent the magnification of the original non-enlarged images; Scale bar for images A-D, 10µm.

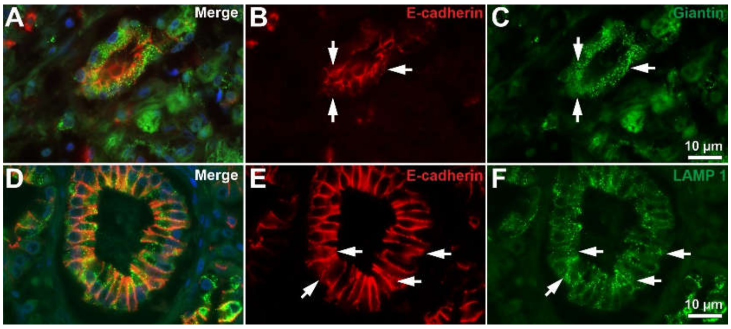

Supplementary Figure S2. Exemplary deconvoluted images of double immunofluorescence for E-cadherin and Giantin (A–C), respectively LAMP1 (D–F) in well differentiated adenocarcinoma epithelium. Colocalizations are indicated (arrows) at both membrane, submembrane and cytoplasmic levels; Scale bar for images A–D, 10µm.

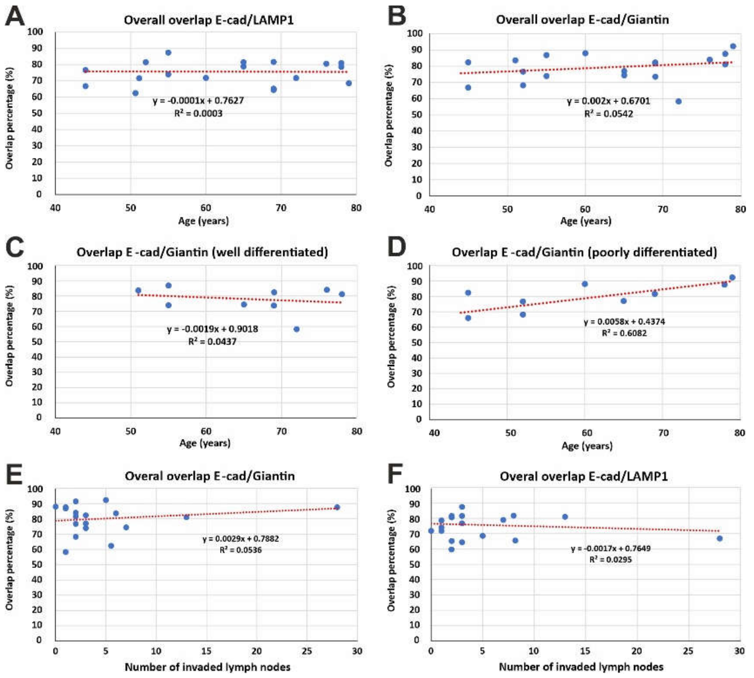

Supplementary Figure S3. Correlations with cancer extension denominators. (A) E-cadherin / LAMP1 global overlapping factors showed no correlation with the age of the patients, while (B) E-cadherin / Giantin global overlapping factors showed a weak correlation with the age of the patients. Dissecting the E-cadherin / Giantin overlapping percentages for (C) well differentiated and (D) poorly differentiated adenocarcinoma cases revealed opposite tendencies for the two degrees of differentiation considered. (E,F) None of the two overlapping factors revealed any correlations with the number of invaded lymph nodes.
